# An acute increase in Left Atrial volume and left ventricular filling pressure during Adenosine administered myocardial hyperaemia: CMR First-Pass Perfusion Study

**DOI:** 10.1186/s12872-023-03230-x

**Published:** 2023-05-11

**Authors:** Pankaj Garg, Wasim Javed, Hosamadin Assadi, Samer Alabed, Ciaran Grafton-Clarke, Andrew J Swift, Gareth Williams, Abdallah Al-Mohammad, Chris Sawh, Vassilios S Vassiliou, Mohammed Y Khanji, Fabrizio Ricci, John P Greenwood, Sven Plein, Peter Swoboda

**Affiliations:** 1 University of East Anglia, Norwich Medical School, Norwich, Norfolk, UK; 2 Norfolk and Norwich University Hospitals NHS Foundation Trust, Norwich, Norfolk, UK; 3 Leeds Institute of Cardiovascular and Metabolic Medicine, University of Leeds, Leeds, UK; 4 Department of Infection, Immunity and Cardiovascular disease, University of Sheffield Medical School and Sheffield Teaching Hospitals NHS Trust, Sheffield, UK; 5 Department of Cardiology, Sheffield Teaching Hospitals NHS Foundation Trust, Sheffield, UK; 6 NIHR Barts Biomedical Research Centre, William Harvey Research Institute, Queen Mary University of London, Charterhouse Square, London, UK; 7 Barts Heart Centre, St Bartholomew’s Hospital, Barts Health NHS Trust, London, UK; 8 Department of Neuroscience, Imaging and Clinical Sciences, “G.d’Annunzio” University of Chieti-Pescara, Chieti, Italy; 9 Norwich Medical School, Norwich Research Park, Norwich, NR4 7UQ UK

**Keywords:** Cardiovascular magnetic resonance, Left ventricular end-diastolic pressure, MRI, Left atrium, Haemodynamics

## Abstract

**Objective:**

To investigate whether left atrial (LA) volume and left ventricular filling pressure (LVFP) assessed by cardiovascular magnetic resonance (CMR) change during adenosine delivered myocardial hyperaemia as part of a first-pass stress perfusion study.

**Methods and results:**

We enrolled 33 patients who had stress CMR. These patients had a baseline four-chamber cine and stress four-chamber cine, which was done at peak myocardial hyperaemic state after administering adenosine. The left and right atria were segmented in the end ventricular diastolic and systolic phases. Short-axis cine stack was segmented for ventricular functional assessment. At peak hyperaemic state, left atrial end ventricular systolic volume just before mitral valve opening increased significantly from baseline in all (91 ± 35ml vs. 81 ± 33ml, *P* = 0.0002), in males only (99 ± 35ml vs. 88 ± 33ml, *P* = 0.002) and females only (70 ± 26ml vs. 62 ± 22ml, *P* = 0.02). The right atrial end ventricular systolic volume increased less significantly from baseline (68 ± 21ml vs. 63 ± 20ml, *P* = 0.0448). CMR-derived LVFP (equivalent to pulmonary capillary wedge pressure) increased significantly at the peak hyperaemic state in all (15.1 ± 2.9mmHg vs. 14.4 ± 2.8mmHg, *P* = 0.0002), females only (12.9 ± 2.1mmHg vs. 12.3 ± 1.9mmHg, *P* = 0.029) and males only (15.9 ± 2.8mmHg vs. 15.2 ± 2.7mmHg, *P* = 0.002) cohorts.

**Conclusion:**

Left atrial volume assessment by CMR can measure acute and dynamic changes in preloading conditions on the left ventricle. During adenosine administered first-pass perfusion CMR, left atrial volume and LVFP rise significantly.

## Introduction

Left atrial (LA) assessment has an established role in assessing left ventricular diastolic function. The LA modulates left ventricular filling and cardiac performance through its role as a reservoir, conduit and booster pump [1]. Cardiovascular magnetic resonance (CMR) cine imaging can accurately quantify LA volume and function [2] and, importantly, predicts clinical outcomes [3–6]. Our recent work has demonstrated that maximum LA volume at left ventricular end-systole, just before the opening of the mitral valve by CMR, is an important marker of left ventricular filling pressure (LVFP) [7, 8].

However, it remains unclear whether LA volumes change acutely due to variations in pre- and after-loading conditions on the ventricle. It is crucial to evaluate the temporal dynamicity of LA adaptability and how it can influence the left ventricular filling pressure. For example, if the pre-loading conditions increased acutely, would the LA volume respond accordingly and vice versa.

Adenosine is a naturally occurring nucleoside that can affect the left ventricular diastolic function. This effect of adenosine on left ventricular diastolic function has been investigated in several studies [9–12]. In some of these studies, adenosine provoked diastolic dysfunction [10].Other studies have debated any effect of adenosine on the left ventricular diastolic function [9]. Whilst others have shown the benefit of adenosine in left ventricular diastolic dysfunction [11]. And hence the effect of adenosine remains controversial on left ventricular diastolic function. It is established that dyspnea occurs during adenosine infusion, and this breathlessness is unrelated to the bronchospasm [13]. This phenomenon again raises the question of whether adenosine causes a rise in left ventricular filling pressure.

We hypothesise that LA volume changes acutely in response to dynamic changes in loading conditions on the ventricle. Hence, the main objective of this study was to understand better how LA volume changes acutely and what impact it has on left ventricular filling pressure during adenosine-administered first-pass perfusion. For this study, we used CMR data previously described [14].

## Methods

### Study population

The study population has been previously described [14]. This was a prospective cohort of patients presenting to the chest pain clinic in a tertiary cardiology unit who were referred on clinical grounds for a stress CMR study to evaluate suspected coronary artery disease. Exclusion criteria were estimated glomerular filtration rate < 30 ml/min/1.73 m^2^ and any contraindication to CMR. We also excluded any patient who did not have a proper four-chamber acquisition which could limit LA assessment. Because of this, 11 patients were excluded from the analysis.

### CMR

All patients had CMR imaging on a 1.5T MRI system (Ingenia, Philips Healthcare, Best, The Netherlands) equipped with dStream technology. A dedicated 28-channel cardiac phased array receiver coil was used. CMR imaging was performed using standard protocols [14, 15].

**Fig. 1:**
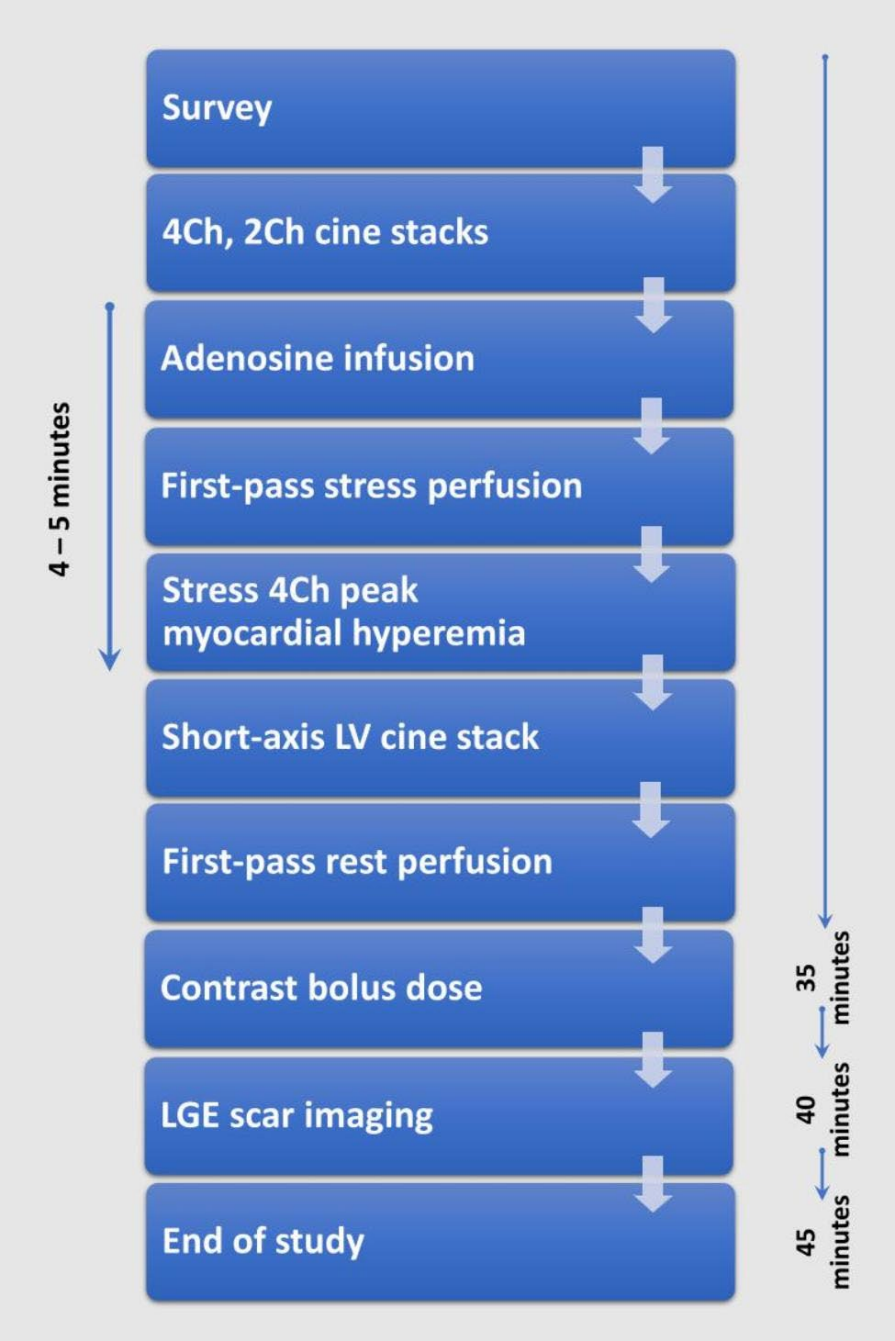
CMR protocol and timeline of different acquisitions. Adenosine infusion was given intravenously infusion at a dose of 140 µg kg − 1 min − 1 for at least 4 min

The CMR protocol included balanced steady-state free precession (bSSFP) baseline cines (4-chamber, 2-chamber, short-axis left ventricular stack) with 30 phases throughout the cardiac cycle, first-past perfusion post adenosine intravenous infusion administration at a dose of 140 µg kg − ^1^ min − ^1^ for at least 4 min and finally, late gadolinium enhancement imaging for scar assessment. First-pass perfusion imaging was immediately followed by the acquisition of a repeat 4-chamber cine for which all parameters were similar to the resting pre-stress 4-chamber view while adenosine was still being infused. The CMR protocol is described in more detail in Fig. 1.

### CMR analysis

All CMR analysis was performed using commercially available software Circle CVI42 version 5.1 (Circle Cardiovascular Imaging Inc., Calgary, Canada). Left ventricular volumes and ejection fraction (EF) were analysed from short-axis cine images using standard methods. The presence of any scar or a significant perfusion defect (> 10% coverage of the left ventricle) was recorded by an expert with > 10 years of experience in CMR. These variables were treated as dichotomous variables.

**Fig. 2:**
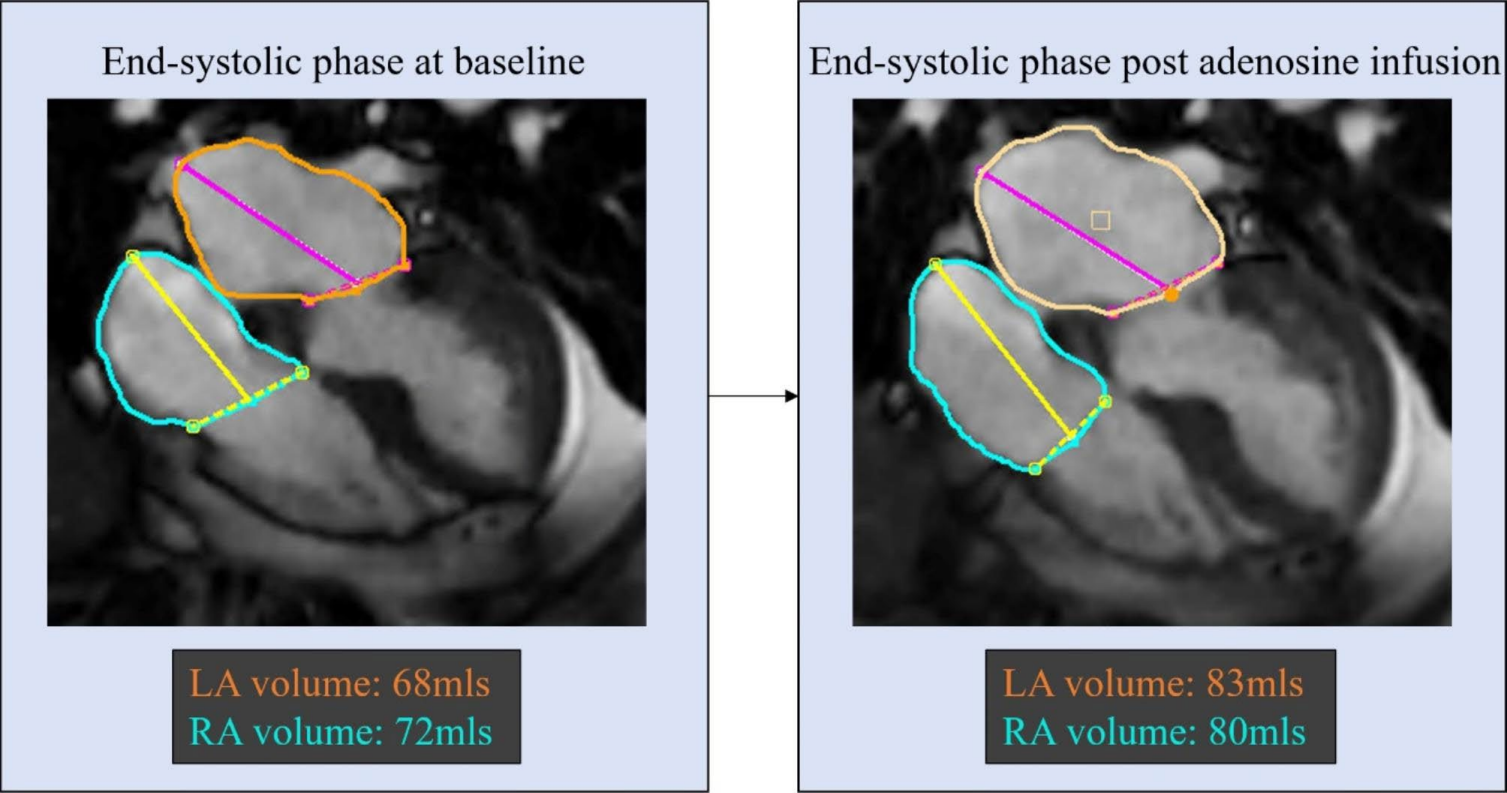
Overview of a case on how left and right atrial volumes were segmented at baseline and peak myocardial hyperemic state after adenosine administration. Both left and right atrial areas were recorded just before the mitral/tricuspid valve opening or ventricular end-systolic phase

For the four-chamber atrial volume analysis, the artificial intelligence module of CVI42 was used. Where necessary, further manual corrections were made. Figure 2 demonstrates how the atrial volumes were assessed at rest and peak myocardial hyperaemia.

### Estimating Pulmonary Capillary Wedge pressure from CMR

Recently, a model to estimate LVFP using CMR-obtained left ventricular mass (LVM) and left atrial volume (LAV) has been developed [8]. This model was derived from 835 subjects referred for further assessment of breathlessness. Multivariable linear regression with backward variable elimination was used to model the relationship between invasive LVFP and CMR-derived measurements. The following equation was used:

CMR-derived LVFP = 6.1352 + (0.07204 x LAV) + (0.02256 x LVM).

Where LVFP is left ventricular filling pressure, LAV is left atrial volume in end-ventricular systole, and LVM is left ventricular mass in end-diastole.

Both atrial ejection fractions were defined from the maximal volume (V_max_) and minimal volume (V_min_) as follows:

left atrial ejection fraction (LAEF) = (LAV_max_ − LAV_min_)/LAV_max_ × 100.

right atrial ejection fraction (RAEF) = (RAV_max_ – RAVmin)/RAV_max_ × 100.

### Statistical analysis

Statistical analysis was performed using MedCalc® Statistical Software version 20.110 (MedCalc Software Ltd, Ostend, Belgium; https://www.medcalc.org; 2022). Continuous variables are presented as mean ± standard deviation or median with interquartile range, as appropriate. Comparisons between rest and stress volumes were performed using paired t-test for normally distributed data or a Wilcoxon signed-rank test for non-parametric data. Categorical variables are summarised by frequency (percentage). Association between continuous variables was quantified using Pearson’s correlation. The two-sided significance level was set at 5%.

### Power calculations

We used previous literature on reproducibility of LA volume to estimate the number of patients needed to be recruited for this study for paired comparison of LA volume pre/post adenosine infusion [16]. We assumed a bias of at least 5mls and wanted to see a mean difference of greater than 8mls with a standard deviation of 15mls. For an alpha of 0.05 and a power of 80%, we needed to recruit a minimum of 30 patients into the study.

## Results

### Patient characteristics

This study enrolled 33 patients with rest and stress four-chamber cine for appropriate atrial segmentation. The mean age of the population was 63 ± 13 years, and 73% were male. From the total cohort, 12 patients (36%) had a history of myocardial infarction. The demographic data for all 33 patients are detailed in Table 1.

**Table 1:** Patient characteristics

Clinical history	*All* *(N* = 33)	*Male* *(N = 24)*	*Female* *(N = 9)*	*P-value*
Age (years)	63 ± 13	62.6 ± 12	64 ± 15	0.78
Current smoker (no. [%])	9 (27%)	8 (33%)	1 (11%)	0.21
Hypertension (no. [%])	9 (27%)	7 (29%)	2 (22%)	0.70
Diabetes Mellitus (no. [%])	10 (30%)	6 (25%)	4 (44%)	0.29
Dyslipidaemia (no. [%])	7 (21%)	6 (25%)	1 (11%)	0.40
Myocardial Infarction (no. [%])	12 (36%)	11 (46%)	1 (11%)	0.07
CABG (no. [%])	4 (12%)	3 (13%)	1 (11%)	0.92
Abnormal ECG (no. [%])	7 (21%)	5 (21%)	2 (22%)	0.93
**CMR characteristics**				
Presence of Infarction (no. [%])	19 (58%)	16 (67%)	3 (33%)	0.09
Perfusion Defect (no. [%])	14 (42%)	12 (50%)	2 (22%)	0.16
LV EDV, (ml/m^2^)	137 ± 41	150.9 ± 37	101.2 ± 25	< 0.0001
LV ESV, (ml/m^2^)	53 ± 32	31.8 ± 9	61.2 ± 33	0.01
LV SV, (ml/m^2^)	82 ± 27	86.3 ± 29	69.3 ± 19	0.11
LV EF, (%)	63 ± 13	68.2 ± 5	61.7 ± 15	0.22
LV Mass (grams)	109 ± 36	120.7 ± 33	76.9 ± 20	< 0.0001
GLS (%)	-19 ± 4	-17.4 ± 4	-21.6 ± 3	< 0.0001
E’ (s − ^1^)	81 ± 40	69.5 ± 22	112.3 ± 61	< 0.0001
A’ (s − ^1^)	82 ± 30	77.1 ± 30	95.9 ± 25	0.11
LAC (ml)	10.5 ± 15	11.2 ± 16	8.8 ± 10	0.69

*Abbreviations A’ myocardial late diastolic velocity, CABG coronary artery bypass grafting, CMR cardiovascular magnetic resonance, E’ myocardial early diastolic velocity, ECG electrocardiogram, EDV end-diastolic volume, EF ejection fraction, ESV end-systolic volume, GLS global longitudinal strain, LAC absolute change in left atrial volume pre and post stress perfusion, LV left ventricular, SV stroke volume.*

The average left ventricular ejection fraction was 63 ± 13%, and 14 patients (42%) had significant perfusion defects suggestive of myocardial ischaemia. In addition, 19 patients (58%) had a ventricular scar due to ischaemic aetiology. The CMR characteristics are summarised in Table 1.

### Gender-related changes

Age (62.6 ± 12 years vs. 64 ± 15 years) differed non-significantly between males and females. No statistical differences were observed between males and females in comorbidities including current smokers (*P* = 0.21), hypertension (*P* = 0.21), diabetes mellitus (*P* = 0.29), dyslipidaemia (*P* = 0.29), previous myocardial infarction (*P* = 0.07), previous coronary artery bypass graft procedure (*P* = 0.92) and having an abnormal electrocardiogram (*P* = 0.93).

Males had a significantly higher LV end-diastolic volume (EDV) (151 ± 37ml/m^2^ vs 101 ± 25ml/m^2^, *P* < 0.0001), LVM (121 ± 33grams vs 77 ± 20grams, *P* < 0.0001), global longitudinal strain (GLS) (-17 ± 4% vs -22 ± 3%, *P* < 0.0001) and a significantly lower LV end-systolic volume (ESV) (32 ± 9ml/m^2^ vs 61 ± 33ml/m^2^, *P* = 0.01) and myocardial early diastolic velocity (E’) (70 ± 22s^− 1^ vs 112 ± 61s^− 1^, *P* < 0.0001) than females. However, no significant changes were observed between the two groups in presence of infarction (*P* = 0.09), perfusion defects (*P* = 0.16), LV stroke volume (SV) (*P* = 0.11), LV ejection fraction (EF) (*P* = 0.22), myocardial late diastolic velocity (A’) (*P* = 0.11) and absolute change in left atrial volume pre and post stress perfusion (LAC) (*P* = 0.69).

### Left atrial volume and function

At peak hyperaemic state, left atrial end ventricular systolic volume just before mitral valve opening increased significantly from baseline in all (91 ± 35ml vs. 81 ± 33ml, *P* = 0.0002), in males only (99 ± 35ml vs. 88 ± 33ml, *P* = 0.002) and females only (70 ± 26ml vs. 62 ± 22ml, *P* = 0.02).Moreover, left atrial ejection fraction was higher at peak hyperaemic state in all (57 ± 12%) than at rest state (53 ± 11%) (*P* = 0.02). However, there were no significant differences between peak hyperemic and rest states in males only (56 ± 13% vs. 52 ± 12%, *P* = 0.05) and females only (59 ± 10% vs. 56 ± 7%, *P* = 0.20) groups. No significant differences were found between left atrial end ventricular diastolic volume at rest and peak hyperaemic state (39 ± 22ml vs. 40 ± 22ml, *P* = 0.50). Similar findings were seen in males only (*P* = 0.80) and females only (*P* = 0.29) cohorts. (Fig. 2**)**.

### Right atrial volume and function

**Fig. 3:**
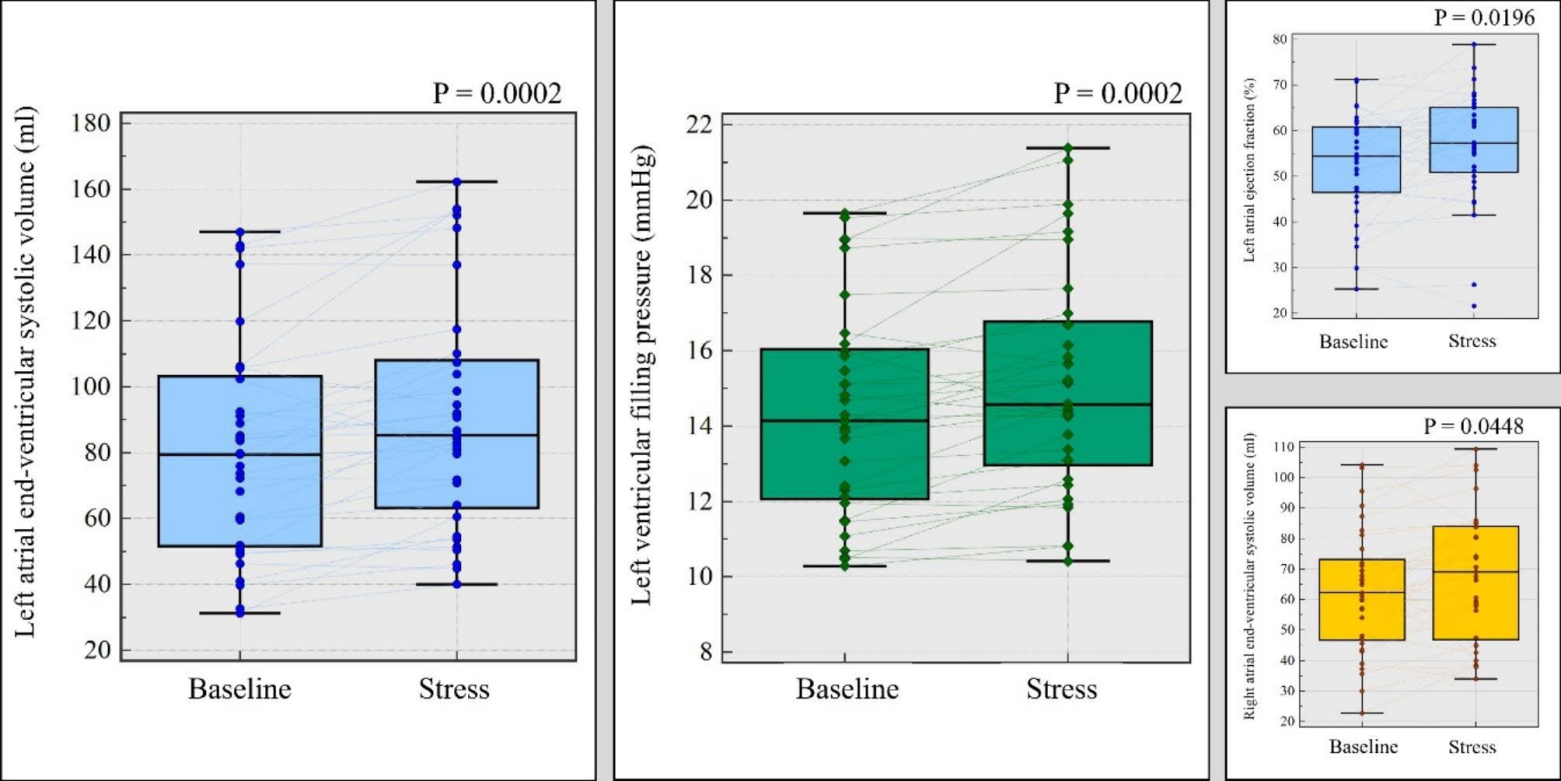
Box plots illustrating the comparison between different CMR parameters at baseline and stress and their significance in all patient cohorts. Predominantly, the left atrial end-ventricular systolic volume increased the most and directly impacted the left ventricular filling pressure, which also correspondingly increased. The left atrial ejection fraction also significantly increased. The increase in right atrial volume was less significant

**Fig. 4:**
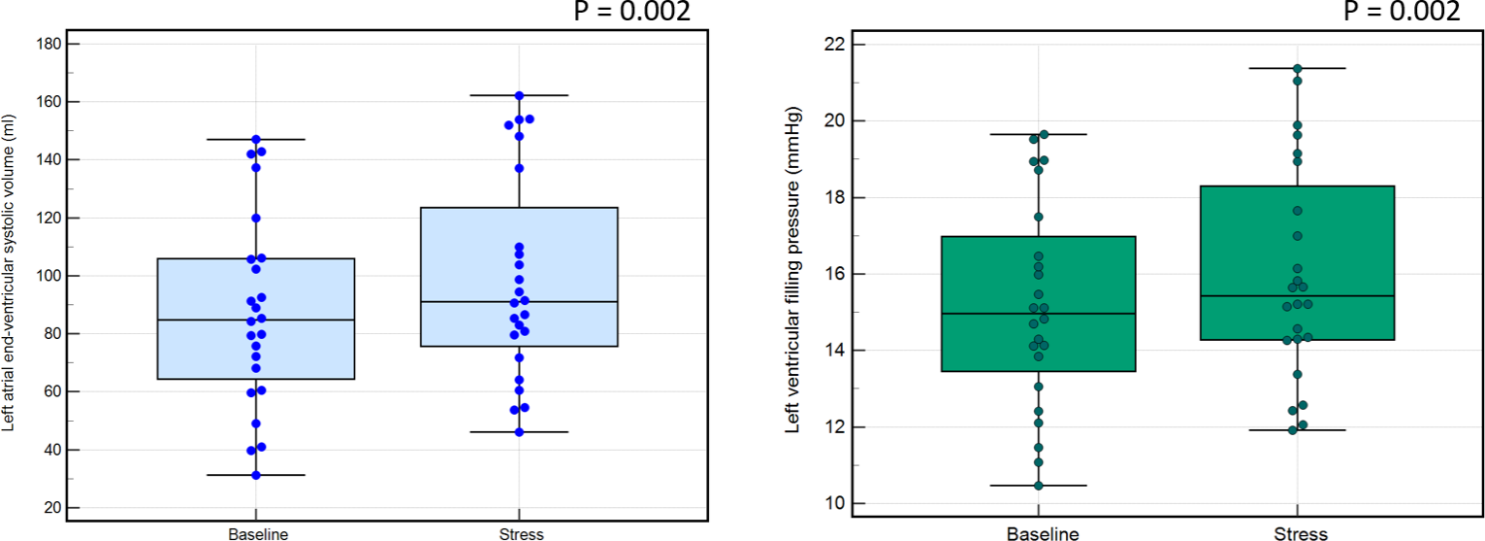
Box plots illustrating the comparison between different CMR parameters at baseline and stress and their significance in males only cohort. Predominantly, the left atrial end-ventricular systolic volume increased the most and directly impacted the left ventricular filling pressure, which also correspondingly increased

**Fig. 5:**
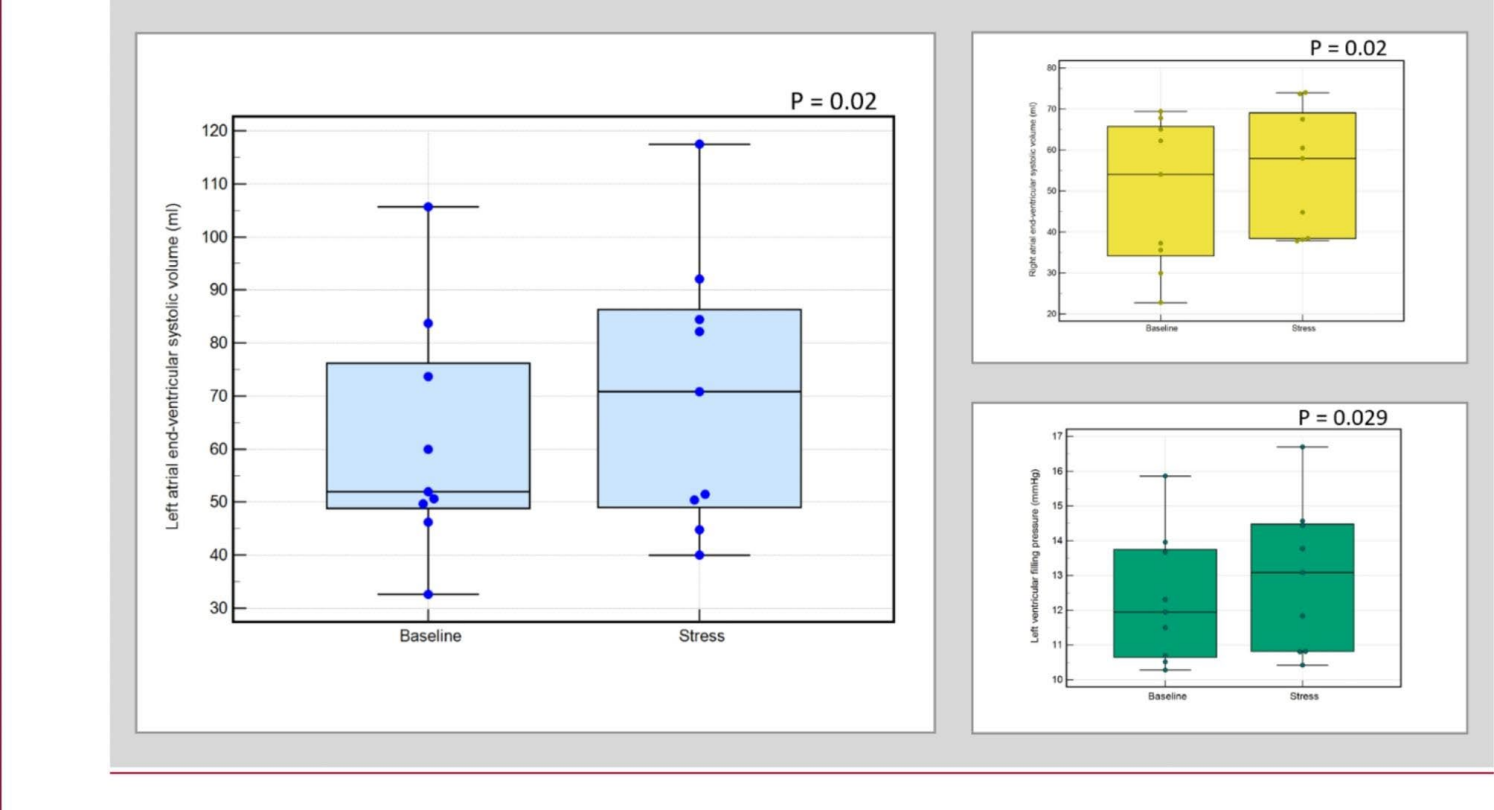
Box plots illustrating the comparison between different CMR parameters at baseline and stress and their significance in females only cohort. Predominantly, the left atrial end-ventricular systolic volume increased the most and directly impacted the left ventricular filling pressure, which also correspondingly increased. The right atrial ejection fraction also significantly increased

The right atrial end ventricular systolic volume increased significantly from baseline in all (63 ± 20ml vs. 68 ± 21ml, *P* = 0.045) and females only group (49 ± 18ml vs. 55 ± 15ml, *P* = 0.02). However, no significant changes were observed in males only group (*P* = 0.14). There were no significant changes observed between peak hyperaemic and rest states when measuring right atrial end ventricular diastolic volume and right atrial ejection fraction (all: 34 ± 16ml vs. 34 ± 14ml, *P* = 0.75; males only: 37.5 ± 17ml vs. 37 ± 14ml, *P* = 0.85; females only: 26 ± 12ml vs. 25 ± 13ml, *P* = 0.63) and (all: 50 ± 15% vs. 47 ± 14%, *P* = 0.21; males only: 49 ± 16% vs. 45 ± 14%, *P* = 0.25; females only: 52 ± 15% vs. 51 ± 13%, *P* = 0.66) respectively (Figs. 3 and 4**and** Fig. 5**)**.

### Left ventricular filling pressure

CMR-derived LVFP (equivalent to pulmonary capillary wedge pressure [PCWP]) increased significantly at peak hyperemic state in all (15.1 ± 2.9mmHg vs. 14.4 ± 2.8mmHg, *P* = 0.0002), females only (12.9 ± 2.1mmHg vs. 12.3 ± 1.9mmHg, *P* = 0.029) and males only (15.9 ± 2.8mmHg vs. 15.2 ± 2.7mmHg, *P* = 0.002) (Figs. 3 and 4**and** Fig. 5**)**.

## Discussion

The key finding of this study is that atrial volumetric assessment by CMR is dynamic and can be used acutely to ascertain loading conditions on the ventricle. We demonstrate that LA volume increases during adenosine administered myocardial hyperaemia.

We have previously demonstrated in a large observational cohort study of 835 patients how CMR assessment of left atrial volume (at end ventricular systolic phase) and left ventricular mass are associated with PCWP measured invasively on the same day [7, 8]. Using the validated LVFP equation from this work, we demonstrated how CMR could provide a complimentary role over the established non-invasive assessment of LVFP. Left ventricular mass (LVM) increases due to a chronic rise in loading conditions on the ventricle [17]. It does not inform acute and dynamic changes, which are also relevant in the routine clinical assessment of patients presenting with heart failure. In this study, we demonstrate that LA volume adds an acute dynamic component to LVFP assessment by CMR.

It is well established that continuous intravenous infusion of adenosine results in sinus tachycardia and coronary-/peripheral- vasodilatation [18]. Importantly, similar to our study, previous invasive haemodynamic studies have also demonstrated a rise in both PCWP and left ventricular end-diastolic pressure (LVEDP). Both PCWP and LVEDP are markers of LVFP. The increase of both LVEDP and PCWP due to adenosine administration indicates that PCWP elevation is not due to an isolated effect on the pulmonary vasculature. Physiologically, if adenosine induces arterial vasodilation, resulting in reduced afterload, LVFP should reduce. Nussbacher et al. have previously proposed that adenosine causes LV diastolic impairment by several mechanisms [19]. Firstly, it may engorge the coronary vasculature with sufficient blood volume to increase ventricular stiffness. Secondly, adenosine could lower contractility, triggering a compensatory rise in cardiac preload. Thirdly, the molecule could prolong the relaxation phase by delaying filling, resulting in elevated diastolic pressures.

Moreover, Nussbacher et al. [19] also noted that adenosine increases preloading conditions on the ventricle. The exact mechanism of this remains speculative. Our finding of an increase in LA end-ventricular systolic volume also suggests that the mechanism involves an increase in preloading conditions on the ventricle with possible LV diastolic dysfunction.

### Limitations

Our study has some limitations. Firstly, left atrial volume assessment was predominantly done in patients with possible ischaemic heart disease. Hence, our results cannot be applied entirely to patients with heart failure. Importantly, in advanced heart failure, where the left atrium has significantly adversely remodelled, it remains uncertain if LA volume changes at all due to changes in loading conditions on the ventricle. Secondly, this study did not recruit hemodynamically unstable patients, further introducing selection bias. Moreover, we did not have echocardiographic findings recorded in the study to demonstrate clinical translation between CMR and echocardiography. Finally, this study was not powered to evaluate changes in LA volume in patients with or without perfusion defect or even the presence of an ischemic scar on late gadolinium enhancement imaging.

## Conclusion

Left atrial volume assessment by CMR can measure acute and dynamic changes in preloading conditions on the left ventricle. During adenosine administered first-pass perfusion CMR, left atrial volume and LVFP rise significantly. Further studies are required to assess whether this can be incremental for diagnosis and prognosis.

## Data Availability

Underlying data: access to the raw images of patients is not permitted since specialised post-processing imaging-based solutions can identify the study patients in the future. The data sets used and/or analysed during this study are available from the corresponding author on reasonable request.

## Abbreviations

CMR: Cardiac magnetic resonance imaging
LA: Left atrium
LAEF: Left atrial ejection fraction
LVFP: Left ventricular filling pressure
LVEDP: Left ventricular end-diastolic pressure
LVM: Left ventricular mass
PCWP: Pulmonary capillary wedge pressure
RAEF: Right atrial ejection fraction
